# Biochemical and Histopathological Alterations in Different Tissues of Rats Due to Repeated Oral Dose Toxicity of Cymoxanil

**DOI:** 10.3390/ani10122205

**Published:** 2020-11-25

**Authors:** Mohamed S. Ahmed, Ahmed H. Massoud, Aly S. Derbalah, Ashraf Al-Brakati, Mohsin A. Al-Abdawani, Hatim A. Eltahir, Tokuma Yanai, Ehab Kotb Elmahallawy

**Affiliations:** 1 Department of Pathology, Faculty of Veterinary Medicine, Kafrelsheikh University, Kafrelsheikh 33516, Egypt; aosayedahmed@yahoo.com; 2 Pesticides Chemistry and Toxicology Department, Faculty of Agriculture, Kafrelshiekh University, Kafr El Sheikh 33516, Egypt; drahmedmassoud2020@gmail.com (A.H.M.); aliderbalah@yahoo.com (A.S.D.); 3 Department of Human Anatomy, College of Medicine, Taif University, P.O. Box 11099, Taif 21944, Saudi Arabia; a.albrakati@tu.edu.sa; 4 Animal Health Research Center, Directorate General of Agriculture and Livestock Research, Ministry of Agriculture and Fisheries, Muscat 117, Oman; mohsinalabdwani@gmail.com (M.A.A.-A.); hatim.eltahir@gmail.com (H.A.E.); 5 Central Veterinary Research Laboratory, Al Amarat, Khartoum 8067, Sudan; 6 Laboratory of Wildlife and Forensic Pathology/Biomedical Science Examination and Research Center, Department of Veterinary Medicine, Faculty of Veterinary Medicine, Okayama University of Science, Okayama 700-8530, Japan; tokumayanai@gmail.com; 7 Department of Zoonoses, Faculty of Veterinary Medicine, Sohag University, Sohag 82524, Egypt; 8 Department of Biomedical Sciences, University of Leon, s/n, 24071 León, Spain

**Keywords:** cymoxanil, rats, liver, brian, testes, cholinesterase, AST, ALT, ALP

## Abstract

**Simple Summary:**

Cymoxanil is a broad-spectrum fungicide used to protect many fruits, vegetables, and field crops against several fungal diseases. Investigating the potential hazards and toxicological effects of this fungicide is very important as cymoxanil can be a major human health concern. The present study investigated the effect of repeated oral doses of cymoxanil on different tissues of treated rats by measuring different biochemical parameters and investigating the histopathological changes. Interestingly, our study reported a dose-dependent effect of cymoxanil that was combined with marked alteration on biochemical enzymes. Moreover, the alteration was combined with marked histopathological changes in various tissues of treated rats, mainly liver, brain, and kidney tissues. Our study collectively reveals that cymoxanil can be a source of major concern for human health with respect to long-term and low dose exposure.

**Abstract:**

Evaluating potential adverse health impacts caused by pesticides is an important parameter in human toxicity. This study focuses on the importance of subchronic toxicity assessment of cymoxanil fungicide in rats with special reference to target biochemical enzymes and histopathological changes in different tissues. In this regard, a 21-day toxicity study with repeated cymoxanil oral doses was conducted. It has been shown that low doses (0.5 mg/kg) were less effective than medium (1 mg/kg) and high (2 mg/kg) doses. Moreover, high dose dose-treated rats showed piecemeal necrosis in the liver, interstitial nephritis and tubular degeneration in the kidneys, interstitial pneumonia and type II pneumocyte hyperplasia in the lungs, gliosis, spongiosis, and malacia in the brain, and testicular edema and degeneration in the testes. Cymoxanil significantly increased AST, ALT, and ALP in serum and liver, indicating tissue necrosis and possible leakage of these enzymes into the bloodstream. Creatinine levels increased, indicating renal damage. Similarly, significant inhibition was recorded in brain acetylcholinesterase, indicating that both synaptic transmission and nerve conduction were affected. Importantly, these histopathological and biochemical alterations were dose-dependent. Taken together, our study reported interesting biochemical and histopathological alterations in different rat tissues following repeated toxicity with oral doses of cymoxanil. Our study suggests future studies on different pesticides at different concentrations that would help urge governments to create more restrictive regulations concerning these compounds’ levels.

## 1. Introduction

Pesticides are a group of chemicals used in agriculture to control diseases and insects and regulate plant growth. Pesticides can be derived from natural compounds or synthesized. Different pesticide applications can be used as herbicides, insecticides, or fungicides [1]. The last few decades have witnessed a marked increase in pesticide use, making them an effective and irreplaceable measure to control plant diseases and pests [2]. The increasing use of pesticides has raised concerns about the risks of harmful residues in crops, impacting human health and environmental safety [3,4]. Nowadays, global pesticide research has mainly assessed pesticide residues and their metabolites in different environments, soil and agricultural products, and dissipation dynamics and half-life [5,6]. However, toxicity studies on animals and humans are rarely conducted [7]. The pesticide industry has been trying to produce lower toxicity pesticides for non-target animals that are more functionally efficient than those currently used; however, pesticides with toxic effects are still being used for specific purposes and can cause metabolic disorders in mammals [8]. Expanding development in the agricultural industry causes excessive pesticide usage, and as a consequence, high levels of pesticide residues are being detected in the environment in different localities in the world and sometimes even accumulate in humans or other mammals [9,10]. Clearly, great attention should be paid to pesticides due to their effects on human and mammalian health [10].

Cymoxanil is a broad-spectrum and systemic fungicide used to protect many fruits, vegetables, and field crops against a wide spectrum of fungal diseases [11]. Cymoxanil belongs to a group of aliphatic nitrogen compounds and acts as a foliar fungicide with protective and curative action. It has contact, local, and systemic activity but also inhibits sporulation. It is authorized for use on potatoes, dry pulses, sunflower seeds, and soybeans that might be fed to livestock [12,13]. Furthermore, it is commonly used to control downy mildew, late blight, and frost in crops [14]. It is noteworthy to state that several previous studies revealed that cymoxanil might have very limited toxicity on mammals and several animals’ species. However, some recent reports about repeated dose toxicity and developmental toxicity of Cymoxanil were recorded during subchronic and chronic toxicity studies; mainly on testes/epididymides of rats and dogs [15,16]. Furthermore, Cymoxanil is toxic to the aquatic organism and causes cardiac developmental toxicity and severe energy deficiency in zebrafish [17]. Mechanistically, cymoxanil toxicity might be due to the downregulation of genes associated with the calcium-signaling pathway and cardiac muscle contraction [17]. Moreover, cymoxanil presents toxins to bees and other aquatic organisms [18,19]. Taken into account, few previous studies documented the residue behavior of cymoxanil. In a previous study, Tebuconazole and cymoxanil have been detected at concentrations of up to 3.2 µg L-1 and 0.9 µg L-1 in surface and ground waters, respectively, from La Rioja region (Spain) that exceed the European Union limit (0.1 μg L−1) [20]. The dissipation and residue of metalaxyl and cymoxanil in pepper and soil were evaluated in another previous study and their residues were below the standard limits of European Union (EU) [3]. The present work aims to evaluate the subchronic toxicity of cymoxanil fungicide in male Sprague–Dawley (SD) rats combined with an assessment of the alteration of biochemical enzymes and histopathological changes in different tissues.

## 2. Materials and Methods

### 2.1. Ethical Statement

Ethical approval was performed as described by the ethical standards of the Faculty of Veterinary Medicine, Kafrelsheikh University, Egypt, and which complies with all relevant Egyptian legislation.

### 2.2. Chemical

Cymoxanil tech, off-white powder, was provided by Saturn Agrochemical Inc, Guangdong 518000, China.

### 2.3. Animals

Sixteen adult male SD rats (N = 16) weighing 100–120 g were obtained from the Faculty of Medicine, Tanta University, and acclimatized for one week before the experiment. All rats were housed in polypropylene cages under standard conditions of 12 h light/dark cycle at 22 ± 2 °C temperature, 30–70% relative humidity, with proper ventilation; standard rat feed and water were provided ad libitum [21].

### 2.4. Animal Treatment

Rats were divided into three treated groups and one control group (four rats per group). Treated rats received a diet containing cymoxanil dissolved in almond oil at a low dose (0.5 mg/kg/day), medium dose (1 mg/kg/day), and high dose (2 mg/kg/day) for 21 successive days. Control group rats were fed a normal diet containing an equal amount of almond oil. Animals were observed for clinical signs of toxicity once a day, and they were weighed every three days over the entire observation period. All animal studies were approved by our Institutional Animal Ethics Committee.

### 2.5. Histopathological Examination

All rats were sacrificed under anesthesia after 21 days. A postmortem examination was performed, and all lesions were recorded. Specimens from all organs, especially the liver, kidneys, brain, lungs, and testes, were taken and kept in neutral buffered formalin 10% for histopathological examination. Specimens were then dehydrated in ascending grades of alcohols, cleared in xylene, embedded in paraffin wax, sectioned at 4 µm, stained with hematoxylin and eosin (HE) and periodic acid-Schiff (PAS) stains, and then examined by light microscopy [22].

### 2.6. Biochemical Determinations

Blood samples were taken by cardiac puncture in vials without anticoagulant for serum collection shortly before the rats were sacrificed under anesthesia. Serum was obtained by blood centrifugation at 3000 rpm for 5 min and kept at −20 °C until use. The livers, kidneys, and brains of treated and control rats were quickly separated on ice-cold 0.8 M sucrose containing 1 mM phenylmethanesulfonyl fluoride (PMSF) using a Miccra D-1-high speed tissue homogenizer to make 10% homogenate (*w*/*v*). The homogenate was centrifuged at 10,000× *g* for 10 min at 4 °C. The supernatant was used as an enzyme source. Aspartate aminotransferase (AST), alanine aminotransferase (ALT), alkaline phosphatase (ALP), and creatinine were determined from the serum and supernatant of the liver and kidney homogenate using commercially available diagnostic kits according to manufactures instructions (LifeSpan Biosciences, USA) as described elsewhere [23,24]. The activity of acetylcholinesterase (AChE) of the liver and kidney homogenate was determined according to the method of Ellman et al. (1961) and Rahman et al. (2000) [25,26]. Briefly, the homogenate mixture of liver and kidney were incubated in 0.1 M Tris-HCl buffer (pH 7.4) and 1.0 mM acetylthiocholine for 15 min at 37 °C combined with shaking. The reaction of this assay was then stopped by addition of a mixture of 52-nitrobenzoic acid and sodium dodecyl sulfate in order to get final concentrations of 0.04 and 0.44%, respectively. Parallel incubations containing 0.01 n&I eserine were used to correct for nonAChE hydrolysis. Measurement of the absorbance was conducted at 412 nm on a Beckman 3600 spectro-photometer. The assay was run in in duplicate.

### 2.7. Statistical Analysis

The treatments were tested by one-way ANOVA using an SPSS statistical software package for Windows version 11.0. Duncan’s multiple range test was used to find out the group effects. *p* ≤ 0.05 was set as the limit of significance.

## 3. Results

### 3.1. Histopathological Changes

#### 3.1.1. Clinical Signs and Postmortem Examination

There were no observed abnormal clinical signs in the groups of rats treated with low-and medium-dose cymoxanil. However, high dose-treated rats showed signs of toxicity after 15 days of treatment in the form of lethargy, dullness, incoordination, and reduced body weight and feed intake; however, the weight loss was not significant. No deaths were observed during the study period among all treated groups. In a postmortem examination, all the organs of rats were free from any macroscopically visible changes.

#### 3.1.2. Histopathological Changes in the Liver

**Figure 1:**
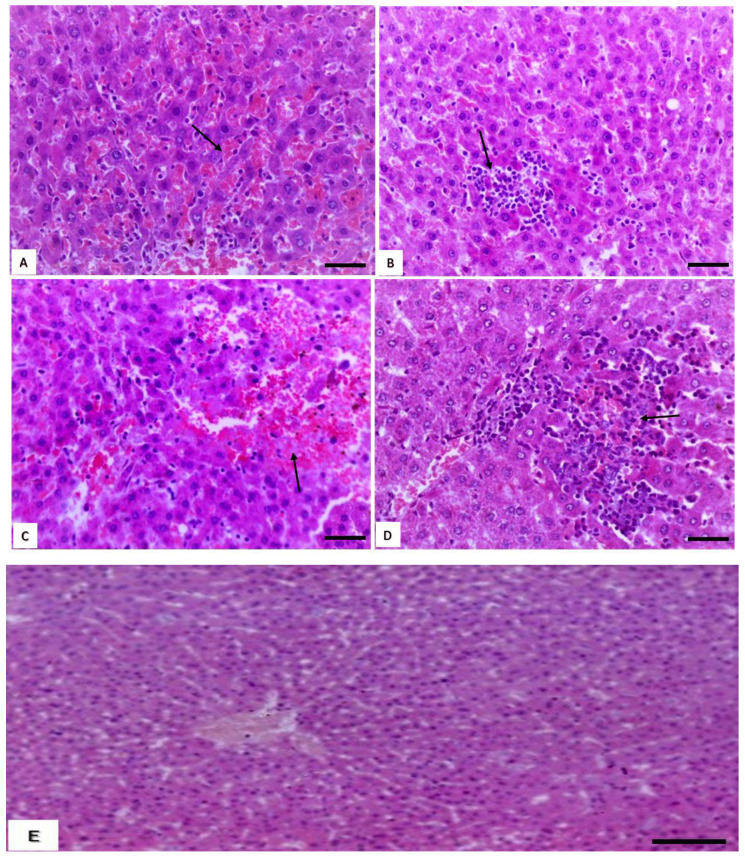
Effect of cymoxanil on the liver. (**A**) Liver of rat treated with low and medium dose showing congestion (arrow) and dilation of the hepatic sinusoids (H&E, bar = 50 µm). (**B**) Liver of rats treated with low and medium dose showing focal hepatocytic necrosis (arrow) with moderate infiltration of mononuclear cells (H&E, bar = 50 µm). (**C**) Liver of rats treated with high dose showing hemorrhages (arrow) (H&E, bar = 50 µm). (**D**) Liver of rats treated with high dose showing extensive piecemeal necrosis (arrow) with infiltration of mononuclear cells within and at the margin of the necrosed areas (H&E, bar = 50 µm). (**E**) Liver of control rats showing hepatic sinusoids in between hepatic cords and they are arranged around central vein (H&E, bar = 50 µm).

The livers of rats in groups treated with low and medium doses of cymoxanil showed congestion and dilatation of hepatic sinusoids (Figure 1A), sinusoidal cell activation mainly with macrophages, and focal hepatocytic necrosis with moderate infiltration of mononuclear cells (Figure 1B). Meanwhile, the livers of high dose-treated rats showed hemorrhages and RBCs scattered within damaged hepatic cords (Figure 1C), and severe inflammatory reactions in the form of extensive piecemeal necrosis with infiltration of mononuclear cells within and at the margin of the necrosed areas (Figure 1D). In addition, the liver of the control group is illustrated in Figure 1E.

#### 3.1.3. Histopathological Changes in the Kidney

**Figure 2:**
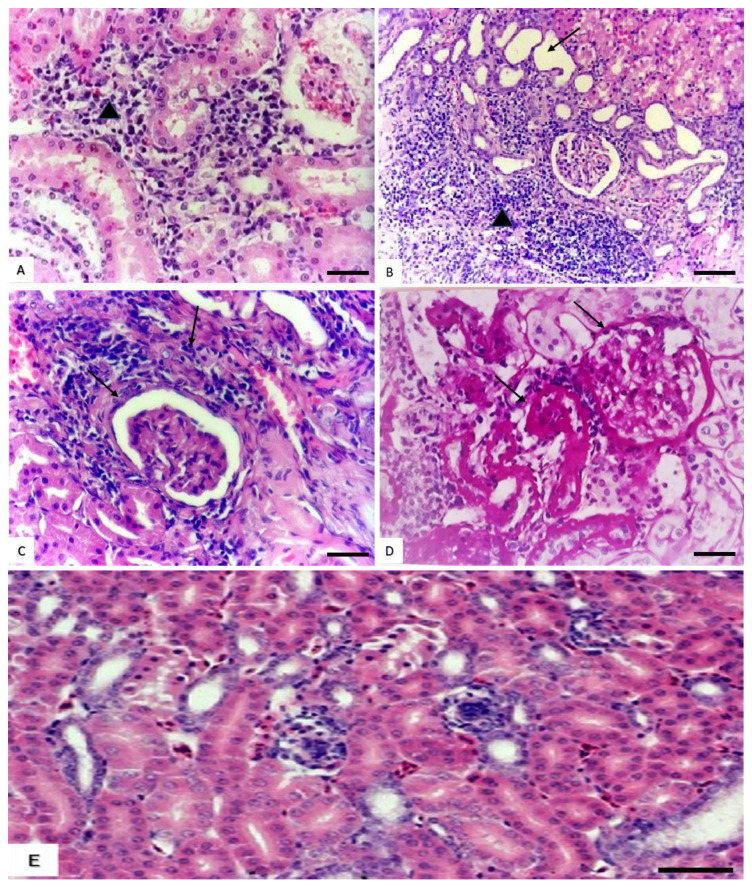
Effect of cymoxanil on the kidneys. (**A**) Kidneys of rats treated with low and medium dose showing slight interstitial nephritis (triangle) with intertubular infiltration of mononuclear cells and degeneration of renal tubule epithelial lining (H&E, bar = 50 µm). (**B**) Kidneys of rats treated with high dose showing severe interstitial nephritis (triangle) and dilatation of the adjacent renal tubules (arrow) (H&E, bar = 100 µm). (**C**,**D**) Kidneys of rats treated with high dose showing thickening and sclerosis of the Bowman capsule and the surrounding renal tubules (arrows) (H&E and PAS respectively, bar = 50 µm). (**E**) Kidneys of control rats showing renal tubules with intact lining epithelium and glomeruli (H&E, bar = 50 µm).

Kidneys of rats in groups treated with low and medium doses exhibited slight interstitial nephritis with intertubular infiltration of mononuclear cells and degeneration of renal tubule epithelial lining (Figure 2A). Meanwhile, in rats treated with high doses, there were marked renal changes in the form of severe interstitial nephritis with massive interstitial mononuclear cell infiltration and dilatation of the adjacent renal tubules (Figure 2B), and thickening and sclerosis of the Bowman capsule and the surrounding renal tubules (Figure 2C,D). On the other hand, kidneys of the control group are expressed in Figure 2E.

#### 3.1.4. Histopathological Changes in the Brain

**Figure 3:**
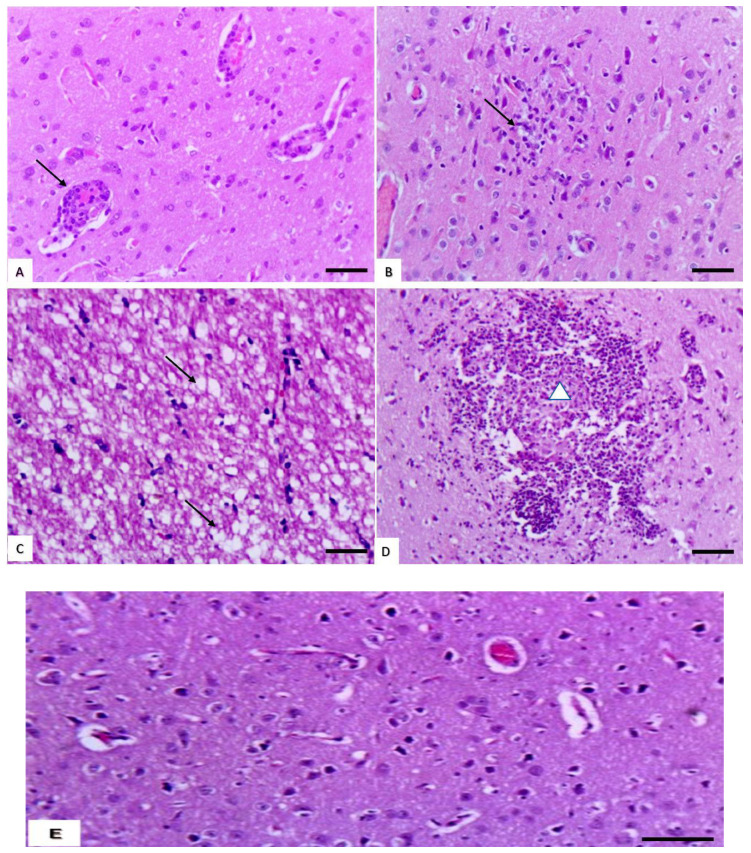
Effect of cymoxanil on the brain. (**A**) Brain of rats treated with medium dose showing perivascular cuff (arrows) (H&E, bar = 50 µm). (**B**) Brain of rats treated with medium dose showing slight gliosis (arrow) (H&E, bar = 50 µm). (**C**) Brain of rats treated with high dose showing cerebral spongiosis (arrow) (H&E, bar = 50 µm). (**D**) Brain of rats treated with high dose showing cerebral malacia (arrow) (H&E, bar = 50 µm). (**E**) Brain of control showing intact neuronal cells and blood vessels surrounded with clear perivascular space in the neuropil of cerebrum (H&E, bar = 50 µm).

There were no obvious changes recorded in the brains of rats treated with cymoxanil at low doses, and the microscopic features of the brains appeared similar to those of control rats. In rats treated with medium doses, the brains showed moderate inflammatory reactions in the form of perivascular cuff (Figure 3A) and slight gliosis (Figure 3B). In rats treated with high doses, the brain lesions became more pronounced in the form of cerebral spongiosis (Figure 3C) and cerebral malacia (Figure 3D). Furthermore, brains of control rats are expressed in Figure 3E.

#### 3.1.5. Histopathological Changes in the Lungs

**Figure 4:**
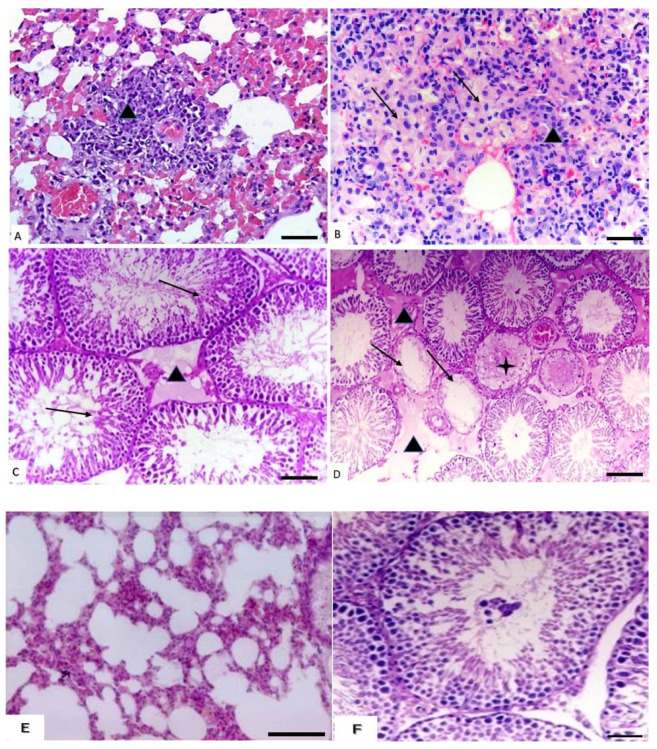
Effect of cymoxanil on the lungs and testis. (**A**) Lungs of rats treated with medium dose showing thickening of the inter-alveolar septa (triangle) (H&E, bar = 50 µm). (**B**) Lungs of rats treated with high dose showing massive interstitial pneumonia (triangle) with pneumocyte type II hyperplasia (arrows) (H&E, bar = 50 µm). (**C**) Testis of rats treated with medium dose showing decrease in the number of spermatogenic layers (triangle) with slight and focal interstitial oedema (star) (H&E, bar = 50 µm). (**D**) Testis of rats treated with high dose showing complete necrosis of the lining epithelium of some seminiferous tubules (triangle) and massive interstitial oedema (star) (H&E, bar = 100 µm). (**E**) Lungs of control rats showing clear alveoli and small interalveolar septa (H&E, bar = 50 µm). (**F**) Testis of control rats showing spermatogenic cells arranged in layers with mature spermatozoa (H&E, bar = 50 µm).

There were no microscopic changes recorded in the lungs of rats treated with low doses, so lungs appeared in the same architecture as control rats. In rats treated with medium doses of cymoxanil, there was thickening of the inter-alveolar septa (Figure 4A). Meanwhile, rats treated with high doses showed massive interstitial pneumonia with mononuclear cells infiltrations were observed in a focal manner with pneumocyte type II hyperplasia (Figure 4B). On the other hand, the histopathological changes in the lung of the control group are illustrated in Figure 4E.

#### 3.1.6. Histopathological Changes in the Testis

No microscopic changes were observed in the testes of rats treated with low doses. Rats treated with medium doses showed a decreased number of spermatogenic cell layers in the seminiferous tubules with slight and focal interstitial edema (Figure 4C). Meanwhile, rats treated with high doses revealed complete necrosis in the epithelial lining of some seminiferous tubules and massive interstitial edema (Figure 4D). On the other hand, the histopathological changes in the testis of the control group are illustrated in Figure 4F.

### 3.2. Biochemical Analysis

#### 3.2.1. Effects on Liver Enzymes

The obtained data showed that the activity of AST, ALT, and ALP gradually increased at different concentration levels (low, medium, and high) in all treated groups with cymoxanil compared to the control group (Table 1).

#### 3.2.2. Effects on Kidney Functions

Regarding kidney functions, creatinine levels increased at all concentration levels of cymoxanil-treated rats (low, medium, and high) compared to the control group (Table 1).

#### 3.2.3. Effects on Brain Function

Regarding acetylcholinesterase activity as a brain function, the obtained results revealed that the activity was slightly decreased in rats treated with low and medium doses. However, it was severely decreased in rats treated with high doses of cymoxanil compared to the control group (Table 1).

## 4. Discussion

Pesticide exposure has been the source of many health problems. The evaluation of potential adverse health impacts caused by pesticides is an important parameter in human toxicity. Taken into account, acute pesticide toxicities have been investigated in many cases. However, limited information is available regarding the medium and long-term toxicity of such compounds. The present study focused on assessing the subchronic toxicity of cymoxanil fungicide with special reference to target biochemical enzymes and histopathological changes in different tissues of male SD rats. As shown in our results, clinical signs and postmortem examinations showed that the low and medium doses were generally asymptomatic. In contrast, high doses caused some changes in the treated rats, such as lethargy, dullness, incoordination, and reduced body weight and feed intake; however, no mortality was observed. We think that the low and medium dose-treated rats were asymptomatic and indicated no observed effect level. However, the high dose-treated rats showed signs of toxicity a short time before the end of the experiment, which indicated that the changes in enzyme activities occurred after the accumulation of a large number of toxicants that resulted in stress conditions in the treated rats [26]. In accordance with the histopathological changes, the liver is a well-known target organ of the toxic effect regarding its function in the biotransformation and excretion of xenobiotics; therefore, it can be used as a toxicity index for various toxic materials [27]. In the present study, slight damage was observed in the liver tissue in both low- and medium-dose-treated groups. In contrast, the hepatic damage was more severe in the form of piecemeal necrosis in rats treated with high doses. These lesions may arise from the toxic effects of cymoxanil, which disturbs the liver’s detoxification mechanisms and induces an inflammatory response comparable with the low- and medium-dose-treated groups [28,29].

It is well known that the liver is the primary organ concerned with detoxification in the body and acts through p450-mediated enzymatic catalysis. We assume that cymoxanil in high doses leads to inhibition of the p450-mediated biocatalysis or adversely affects the mitochondrial membrane transport system of hepatocytes in the liver of treated rats; and thus induces hepatocyte damage and cell death, which is in agreement with previous reports [10,28,30]. Taken into account, kidneys are responsible for eliminating metabolic waste products and controlling the amount and composition of body fluids [31]. The induced histopathological changes in the kidneys of treated rats in the current study corroborated several previous studies [32,33], which reported that there were dose-dependent renal changes in the form of marked tubular dilation, hydropic degeneration in the tubular epithelial lining, cloudy swelling, moderate congestion, and hemorrhage in the cortex and medulla in the kidneys of rats treated with different doses of the organophosphate pesticide fenitrothion. In accordance with histopathological changes in the brain, the observed changes indicated that the brain damage was dose-dependent. In the present study, the low-dose-exposed rats were mostly asymptomatic. However, medium- and high dose-treated rats exhibited signs and symptoms of toxicity, which indicated the stress condition of these rats [7]. Many pesticides kill insects by targeting their nervous system and can cause neurotoxic effects in mammals [34]. We think that cymoxanil interferes with chemical neurotransmission or ion channels and causes reversible neurotoxic effects such as neuritis and gliosis, corroborating previous studies [35,36]. The observed spongiosis may be due to myelin damage or destruction. The brain is highly susceptible to toxicity because it contains relatively low levels of anti-oxidative stress enzymes and high myelin-associated contents, making it vulnerable to the propagation of the peroxidative process [37]. Myelin helps in transmitting signals along the nerves, and the loss of myelin causes nerve damage in neurological diseases [38]. It should be stressed that there are only a few studies concerning the respiratory effects of workers involved with pesticides, and some pesticides may result in pulmonary function impairment [39]. We think that the resulted pulmonary lesions in the present study were due to the breakdown of the alveolar epithelial/endothelial barrier and the exudative inflammatory infiltrate into the lungs; this result corroborated previous reports [40]. However, a previous study mentioned that the pathophysiological processes leading to these inflammatory reactions are unclear, but the pulmonary toxicity can induce acute inflammatory reactions with different features [41]. In the present study, the rats treated with medium doses revealed decreased spermatogenic layers and slight and focal interstitial edema. These lesions became more severe in rats treated with high doses that showed necrosis and edema in the seminiferous tubules and interstitial tissue. These results agree with several previous studies [42,43,44], which reported decreased spermatogenic cell number in the testes and inhibition of spermatogenesis in rats treated with phosphorothionate, acephate, and methyl parathion, respectively.

Transaminases and phosphatases are important critical enzymes in biological processes and are considered specific biochemical indicators of liver damage [28,45]. AST and ALT enzymes are important to the metabolism of cellular nitrogen, liver glucose, and the oxidation of amino acids. They are found in hepatocytes, the heart, kidneys, skeletal muscles, and the pancreas. Increasing the activity of these enzymes plays an important role in amino acid oxidation or transformation during gluconeogenesis [46]. Alkaline phosphatase plays an integral role in glycogen metabolism in the liver by stimulating glucose synthesis to overcome energy required during stress conditions. So, alkaline phosphatase has been used to indicate liver damage and as a hallmark for liver dysfunction [11,45,47]. The elevation of ALT activity appears to reflect acute hepatic disease more specifically than AST values. The activity of either enzyme, particularly AST, may also be elevated in extrahepatic disease. However, the elevation of AST and ALT and the elevation of ALP activities may reflect some necroinflammatory disease of the liver [48]. In the present study, we found that all cymoxanil-treated rat groups had significantly higher AST, ALT, and ALP levels than the control rats. The elevation of liver enzymes might be due to hepatocyte membrane damage; hence, several enzymes in the hepatocyte cytosol are released into the bloodstream, and this is positively correlated with the observed histopathological changes in the liver tissue and corroborates the findings of several previous studies [49,50,51], which recorded the same results in rats treated with organophosphorus compounds. As depicted in our results, the increase of creatinine levels in rats treated with cymoxanil compared to control rats is assumed to be due to impairment of the glomerular function and tubular damage in the kidneys. Moreover, the histopathological changes in kidney tissue, such as inflammation and sclerosis, confirm this explanation. These results agree with some previous reports [52,53], which mentioned that creatinine excretion is entirely dependent on the process of glomerular filtration. Importantly, acetylcholinesterase is a neurotransmitter with stimulatory and inhibitory effects on muscles and has been considered a common indicator for altering brain neural function [7]. Acetylcholinesterase is also considered an index of cellular activity and can be considered a useful toxicological tool [54]. In the present study, AChE decreased in the rats of all treated groups, which was marked in high dose-treated rats. This inhibition led to the accumulation of acetylcholine at cholinergic synapses. The excess acetylcholine causes constant acetylcholine receptor triggering, resulting in an acute cholinergic crisis [35,36]. We suggest that the decrease in the activities of these enzymes might be due to the interaction of cymoxanil with AChE causing alterations in cell membrane permeability and inducing inhibition. These results agree with the findings of several researchers who reported that the most prominent clinical effects of organophosphorus poisoning are related to their inhibition of blood cholinesterase activity [55,56].

## 5. Conclusions

Given the above information, the changes observed by cymoxanil in target biochemical enzymes and histopathological alterations in various treated rat tissues were dose-dependent. The disruption in biochemical parameters after cymoxanil treatment was confirmed by clearly histopathological changes in the treated rats’ livers, brains, and kidneys. The reported results did not show high cymoxanil toxicity at the used concentration levels, but it indicated that cymoxanil could be a source of major concern for human health with respect to long-term and low dose exposure. Our study suggests similar future studies on different pesticide compounds at different low concentrations, close to environmental levels. The recorded toxicity of cymoxanil and possibly other compounds at these low concentration levels could encourage governments to create more restrictive regulations about these compounds’ levels.

## Figures and Tables

**Table 1:** Effect of cymoxanil at different concentration levels on some biochemical parameters in rats.

Dose (mg/kg/bw)	ALT Activity	AST Activity	ALP Activity	ACHE Activity	Creatinine (mg/di)
0.5	23.96 ± 1.68 ^c^	17.36 ± 1.13 ^c^	4.158 ± 0.48 ^c^	0.882 × 10^−4^ ± 0.015 ^a^	0.772 ± 0.007 ^b^
1	22.75 ± 1.34 ^bc^	18.25 ± 0.95 ^b^	4.542 ± 0.32 ^b^	0.752 × 10^−4^ ± 0.013 ^b^	0.780 ± 0.014 ^b^
2	33.28 ± 2.31 ^a^	25.51 ± 1.09 ^a^	5.360 ± 0.54 ^a^	0.369 × 10^−4^ ± 0.012 ^c^	0.957 ± 0.015 ^a^
Control	17.76 ± 0.94 ^d^	13.44 ± 1.45 ^d^	2.798 ± 0.30 ^d^	946 × 10^−4^ ± 17.34 ^a^	0.322 ± 0.013 ^c^

All data was expressed as mean ± S.D (number of replicates = 3). Means within the same column (in each parameter) carrying different superscripts (a, b, c, d) is significantly different (*p* < 0.05).
